# Altered Brain Morphometry in Cerebral Small Vessel Disease With Cerebral Microbleeds: An Investigation Combining Univariate and Multivariate Pattern Analyses

**DOI:** 10.3389/fneur.2022.819055

**Published:** 2022-02-23

**Authors:** Jing Li, Hongwei Wen, Shengpei Wang, Yena Che, Nan Zhang, Lingfei Guo

**Affiliations:** ^1^Department of Radiology, Beijing Friendship Hospital, Capital Medical University, Beijing, China; ^2^Key Laboratory of Cognition and Personality (Ministry of Education), Chongqing, China; ^3^School of Psychology, Southwest University, Chongqing, China; ^4^Research Center for Brain-Inspired Intelligence, Institute of Automation, Chinese Academy of Sciences, Beijing, China; ^5^University of Chinese Academy of Sciences, Beijing, China; ^6^Department of Clinical Laboratory, Shandong Provincial Hospital Affiliated to Shandong First Medical University, Jinan, China; ^7^Department of Radiology, Shandong Provincial Hospital Affiliated to Shandong First Medical University, Jinan, China

**Keywords:** cerebral small vessel disease, cerebral microbleeds, gray matter volume, white matter volume, multivariate pattern analysis

## Abstract

**Purpose:**

The objective of this study was to evaluate whether altered gray matter volume (GMV) and white matter volume (WMV) are associated with the presence of cerebral microbleeds (CMBs) in cerebral small vessel disease (CSVD).

**Materials and Methods:**

In this study, we included 26 CSVD patients with CMBs (CSVD-c), 43 CSVD patients without CMBs (CSVD-n) and 39 healthy controls. All participants underwent cognitive assessment testing. Both univariate analysis and multivariate pattern analysis (MVPA) approaches were applied to investigate differences in brain morphometry among groups.

**Results:**

In univariate analysis, GMV and WMV differences were compared among groups using voxel-based morphometry (VBM) with diffeomorphic anatomical registration through exponentiated lie algebra (DARTEL). Compared to healthy controls, the CSVD-c group and CSVD-n group showed significantly lower GMV than the control group in similar brain clusters, mainly including the right superior frontal gyrus (medial orbital), left anterior cingulate gyrus, right inferior frontal gyrus (triangular part) and left superior frontal gyrus (medial), while the CSVD-n group also showed significantly lower WMV in the cluster of the left superior frontal gyrus (medial). No significant GMV or WMV differences were found between the CSVD-c group and the CSVD-n group. Specifically, we applied the multiple kernel learning (MKL) technique in MVPA to combine GMV and WMV features, yielding an average of >80% accuracy for three binary classification problems, which was a considerable improvement over the individual modality approach. Consistent with the univariate analysis, the MKL weight maps revealed default mode network and subcortical region damage associated with CSVD compared to controls. On the other hand, when classifying the CSVD-c group and CSVD-n group in the MVPA analysis, we found that some WMVs were highly weighted regions (left olfactory cortex and right middle frontal gyrus), which hinted at the presence of different white matter alterations in the CSVD-c group.

**Conclusion:**

Our findings not only suggested that the localized alterations in GMV and WMV appeared to be associated with the pathophysiology of CSVD but also indicated that altered brain morphometry could be a potential discriminative pattern to detect CSVD at the individual level.

## Introduction

Cerebral small vessel disease (CSVD) refers to different pathologic changes involving small intracranial blood vessels, including small arteries, arterioles, capillaries, and small veins (1). CSVD plays a crucial role in lacunar stroke and brain hemorrhages and is a leading cause of functional loss and dementia in the elderly population (2). Neuroimaging is considered the gold standard for detecting CSVD, and the key neuroimaging markers include small cerebral infarctions, lacunes, white matter hyperintensities (WMH), enlarged perivascular spaces (PVS), cerebral microbleeds (CMBs), and brain atrophy (3). Among these, CMBs have been recognized to play a synergistic role in both cerebrovascular and neurodegenerative pathology occurring in the aging brain (4). CMBs are well-demarcated, hypointense, rounded lesions on magnetic susceptibility-sensitive sequences of magnetic resonance imaging (MRI). These hemosiderin-rich lesions form when the heme iron in red blood cells leaks out from brain micro-vessels and are then sequestered by macrophages for storage (5). Several large clinical studies have established an association of CMBs with vascular and systemic inflammation (6) as well as with cognitive decline in patients with vascular dementia and Alzheimer's disease (AD) (7) and in elderly subjects (8).

As one of the important neuroimaging markers of CSVD, CMBs have a significant impact on the cognitive function of patients with CSVD (9). Histopathologic studies have shown that the presence of CMBs indicates widespread damage in arterioles by hypertension, amyloid deposition and surrounding gliosis, infarction, or even necrosis, resulting in microstructural damage to the surrounding white matter. Therefore, CMBs may disrupt white matter tracts involved in cognitive function, leading to damage to neural networks (10). Therefore, cognitive impairment in patients with CMBs is thought to correlate with brain damage in white matter and gray matter structures, and exploring the relationship between CMBs and cerebral morphological changes with new techniques is a current hotspot for CSVD research.

WMH and lacunar infarct correspond to pathophysiological changes including neuron death, demyelination, and axon loss and these subcortical lesions may cause degenerative cortical atrophy in frontal and temporal area (11). Therefore, many studies reported reduced GMV and WMV in CSVD patients (11–13). However, few studies have investigated the relationship between brain volume and CMBs, particularly the relationship between CMBs and WM. A previous study findings speculated that higher CMBs were associated with WM atrophy but not associated with GM atrophy and concluded this caused by CMBs interrupting brain network connectivity (14). Another study found that CSVD subjects with deep or infratentorial CMBs had a lower amygdala GMV than the CSVD subjects with no CMBs after adjusting for age, sex, and total intracranial volume (TIV), although this significance was no longer present after further adjustment for other vascular risk factors (hypertension, diabetes, cigarette smoking, alcohol consumption, body mass index (BMI), and chronic kidney disease (CKD). On the other hand, subjects with strictly lobar CMBs had larger total, frontal, and occipital WMVs than the CSVD subjects with no CMBs (15). The results of previous studies were inconsistent in the relationship between CMBs and WMV morphological changes, but these results both gave us the hint that there existed WM changes in CSVD patients with CMBs compared with CSVD patients without CMBs. Meanwhile, no healthy subjects were included in these studies, and the brain morphological alterations between CSVD patients and healthy controls had not yet been investigated.

Using traditional mass-univariate analyses to quantify the alterations in GM or WM density or volume between groups in a voxel-wise manner has an important limitation in that mass-univariate analyses only aim to test whether there are any effects in one or more brain regions rather than to test whether the effects are large enough to have translational importance for clinical utility (15). Recently, researchers have developed a growing interest in applying multivariate pattern analysis (MVPA) to develop neuroimaging biomarkers for clinical diagnoses of brain diseases (16). MVPA is a promising machine-learning-based pattern recognition technique that can be used to classify neuroimaging data by discriminating between two or more classes (or groups). Relative to traditional univariate analysis, MVPA has two advantages. First, MVPA takes the intercorrelation between voxels into consideration and thus might be more sensitive in detecting subtle and spatially distributed alterations. Second, MVPA allows statistical inferences at the single-subject level and thus could be used to make diagnostic decisions regarding individual patients (17). MVPA and machine-learning methods have been successfully applied in the risk stratification of various diseases along with CSVD, including WMH and enlarged PVS (18–20). However, no study has investigated the utility of MVPA with brain morphometric features for the three binary classification problems in CSVD patients with CMBs (CSVD-c), CSVD patients without CMBs (CSVD-n) and healthy controls.

Therefore, we aimed to apply both mass-univariate and multivariate pattern analysis methods to evaluate brain morphological alterations in a relatively large sample of CSVD patients with or without CMBs. We hypothesized that (1) the CMBs accompanying CSVD will bring about specific brain morphological changes; (2) MVPA analysis would potentially be able to discriminate individual patients with CSVD from healthy controls; and (3) information will be provided on neurobiological changes that will potentially help to elucidate the potential pathogenesis of CSVD.

## Materials and Methods

### Subjects

This was a cross-sectional study approved by the institutional review board of Shandong Provincial Hospital Affiliated to Shandong First Medical University. Between December 2018 and August 2019, 26 CSVD patients with CMBs (age: 67.08 ± 6.19 years; 10 females) and 43 CSVD patients without CMBs (age: 66.79 ± 5.19 years; 22 females) were recruited. We also included 39 healthy subjects (age: 63.90 ± 8.98 years; 22 females) in our study. The inclusion criteria for CSVD patients included diagnosis of recent small subcortical infarct, lacunes of presumed vascular origin, WMH of presumed vascular origin, enlarged PVS, CMBs, and brain atrophy, based on current MRI consensus standards (3). The severity of WMH was assessed using the Fazekas scale. The scale grades the severity from 0 to 3grade. 0 represents occasional or non-punctate WMH; grade 1, multiple punctate WMHs; grade 2, bridging of punctate WMHs leading to confluent lesions; and grade 3, widespread confluent WMH (21). The total CSVD disease burden was assessed by amended CSVD score (0–7 scale; scores calculated based on the severity of CMBs, lacunes, and WMH) that was recently recommended for predicting cognitive decline (22).

### Image Acquisition

All subjects were imaged on a MAGNETOM Skyra 3.0 T MR scanner (Siemens Healthcare, Erlangen, Germany) using a product 32-channel head coil for signal reception. The brain scanning protocol consisted of a 3D T1-weighted (T1W) magnetization-prepared rapid gradient echo (MPRAGE) sequence for anatomical structure (repetition time (TR) = 7.3 ms, echo time (TE) = 2.4 ms, inversion time (TI) = 900 ms, flip angle = 9°, isotropic voxel size = 1 mm^3^) and a 3D multi-echo gradient echo (mGRE) sequence for quantitative susceptibility mapping (QSM) (TR = 50 ms, first TE = 6.8 ms, TE interval = 4.1 ms, number of echoes = 10, flip angle = 15°, voxel size = 1 ×1 ×2 mm^3^). In addition, T2-weighted (T2W) turbo spin echo, T2W fluid-attenuated inversion recovery (FLAIR), diffusion-weighted, and susceptibility-weighted imaging (SWI) scans were acquired to detect brain abnormalities. Before the scan, all participants remained in a normal state of respiration and heart rate. All participants were required to be awake and quietly breathing until the end of the scan.

### Diagnosis of CMBs in CSVD Patients

Through the conventional MRI sequence and SWI images, small subcortical infarct, lacune of presumed vascular origin, WMH of presumed vascular origin, PVS, CMBs, and brain atrophy were diagnosed by a senior neuroradiologist. CMBs are small (generally 2–5 mm in diameter) hypointense lesions that are visible on paramagnetic-sensitive MRI sequences such as T2^*^-weighted gradient-recalled echo (GRE) or susceptibility-weighted sequences and are most commonly located in the cortico-subcortical junction and deep gray or white matter in the cerebral hemispheres, brainstem, and cerebellum (23–25).

### Cognitive Assessments

All participants underwent the Montreal Cognitive Assessment (MoCA) Beijing version (www.mocatest.org), which is a one-page 30-point test administered in 10 min (26). The optimal cutoff for detecting cognitive impairment was 13/14 points for illiterate individuals, 19/20 for individuals with 1–6 years of education, and 24/25 for individuals with 7 or more years of education (27). In addition, a variety of executive functions, including flexibility, working memory and inhibition, were assessed. Briefly, these tests included the following: the Rey auditory verbal learning test (AVLT) for assessing verbal memory abilities (28); the symbol digit modalities test (SDMT) for evaluating attention and information processing speed (29); the trail-making test (TMT) for evaluating attention, information processing speed, visual search and motor coordination (30); and the Stroop color-word test (SCWT) (31). The test implementer was professionally trained and qualified and had no knowledge of the subject grouping.

### VBM-DARTEL Processing

**Figure 1:**
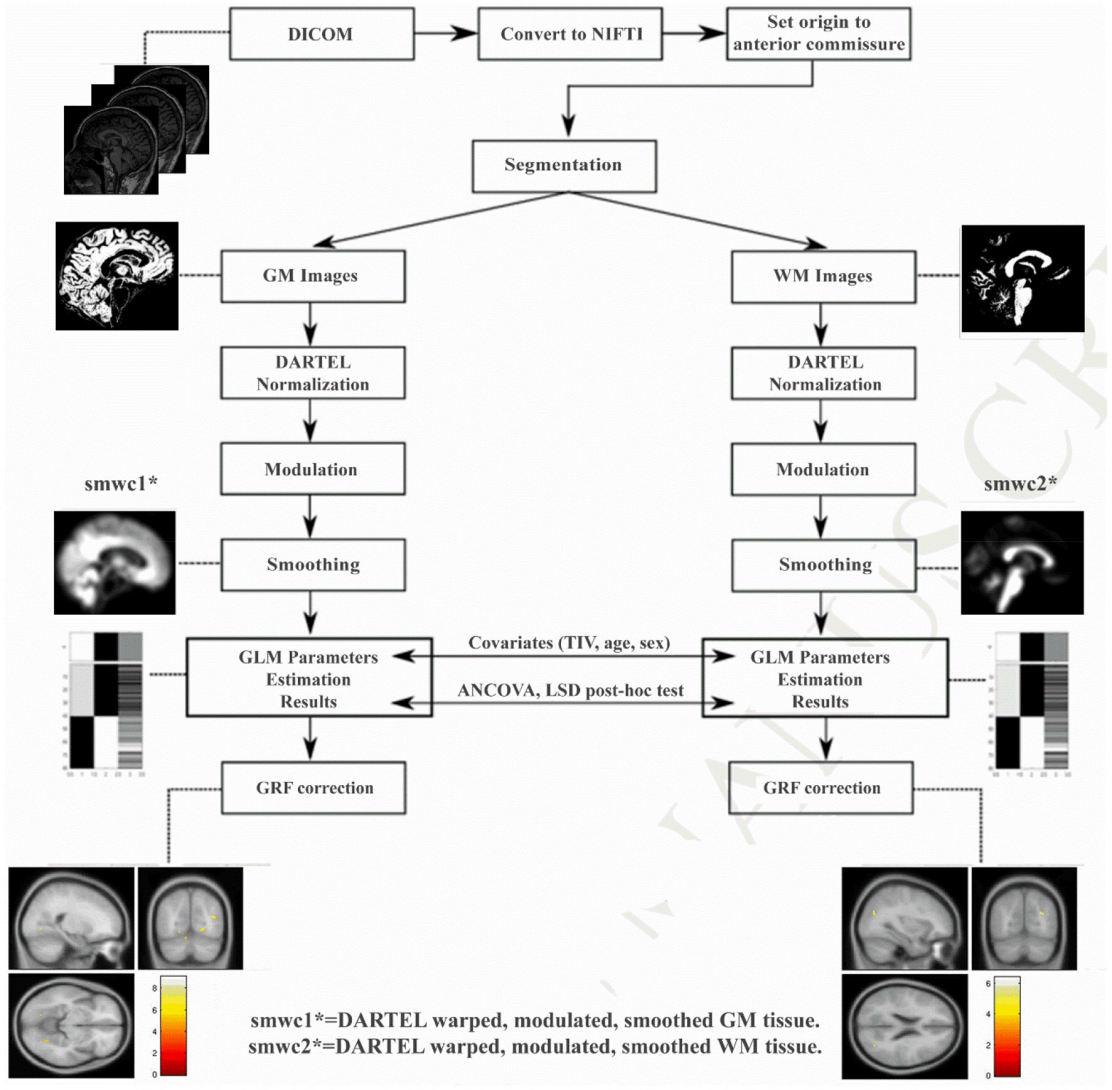
The processing pipeline of VBM-DARTEL analysis using Statistical Parametric Mapping software. DARTEL, diffeomorphic anatomical registration through exponentiated lie algebra; GRF, Gaussian random field.

After data acquisition, 3D T1W image processing was performed using VBM with Diffeomorphic Anatomical Registration Through Exponentiated Lie Algebra (DARTEL) (32) based on the statistical parametric mapping (SPM8, http://www.fil.ion.ucl.ac.uk/spm) toolbox (pipeline shown in Figure 1). DARTEL is a fully deformable registration and normalization method that provides precise inter-subject alignment throughout the iterative unified model. First, all the 3D T1W images were aligned to conventional AC-PC space using manually identified landmarks, including the anterior commissure (AC), the posterior commissure (PC), and the mid-sagittal plane. Then, the aligned images were segmented into GM, WM and cerebrospinal fluid (CSF) in native space with unified segmentation using the *New Segment* tool in SPM (16). Afterward, all the segmented GM and WM images were rigidly transformed to produce a series of aligned GM and WM images. The study-specific GM templates were then created by the DARTEL algorithm with the aligned serial images from all the subjects. During the template creation process, all aligned images were warped to the template, yielding a series of flow fields, which parameterized the deformation. After normalization and modulation, the modulated data were transformed into Montreal Neurological Institute (MNI) space. Finally, the gray matter volume (GMV) and white matter volume (WMV) and partitions were smoothed with an isotropic Gaussian kernel of 8-mm full-width at half-maximum (FWHM).

### Univariate Analysis

To compare GMV and WMV and identify abnormalities among the three groups, one-way analysis of covariance ANCOVA was performed with age, sex and TIV as covariates using the *DPABI* toolbox. For *post-hoc* tests, the least-significant difference (LSD) method was applied, and the corrected *p*-values for comparing group means of any pairs were calculated (33). Then, the p maps were converted to Z maps, and using the Z maps, we performed Gaussian random field (GRF) correction (34) to correct for multiple comparisons. The statistical threshold was set at a voxel-level *p* < 0.001 with a cluster-level *p* < 0.05 (two-tailed) in the *DPAB*I toolbox (33). All coordinates are reported in MNI space. Brain regions with significant intergroup differences in GMV were defined as regions of interest (ROIs), and the mean GMV and WMV of these ROIs were extracted from CSVD patients. Pearson's correlations between mean GMV and clinical parameters were calculated using SPSS Version 24.0 (SPSS Inc, Chicago, IL, USA), and significance was set to *p* < 0.05.

### Multivariate Pattern Analysis

MVPA was carried out to classify different groups based on GMV and WMV maps and investigate unique information that may be overlooked by univariate approaches. The Pattern Recognition Neuroimaging Toolbox (PRo-NTo) (35) was used to implement a binary classifier based on the multiple kernel learning (MKL) approach, which models the whole brain as a combination of regional patterns and therefore learns the contribution of different brain regions to the classification model (16). As an optimized MKL technique called “simple MKL” implemented in PRoNTo assumes sparsity in the kernel combination, this technique selects only a subset of brain regions to perform the classification, and the remaining regions have a null contribution to the model. Regions were defined using the automated anatomical labeling (AAL) atlas (36), which splits the brain into 90 cortical and subcortical regions (detailed shown in Supplementary Table S1). For each region and each modality, a linear kernel was computed based on the regional pattern containing all voxels within the region, and MKL was used to combine multiple (modalities × number of regions) kernels. Considering that the number of voxels varies among brain regions, the kernels were mean-centered and normalized using standard kernel operations implemented in PRoNTo. Age, sex and TIV were included as covariates.

**Figure 2:**
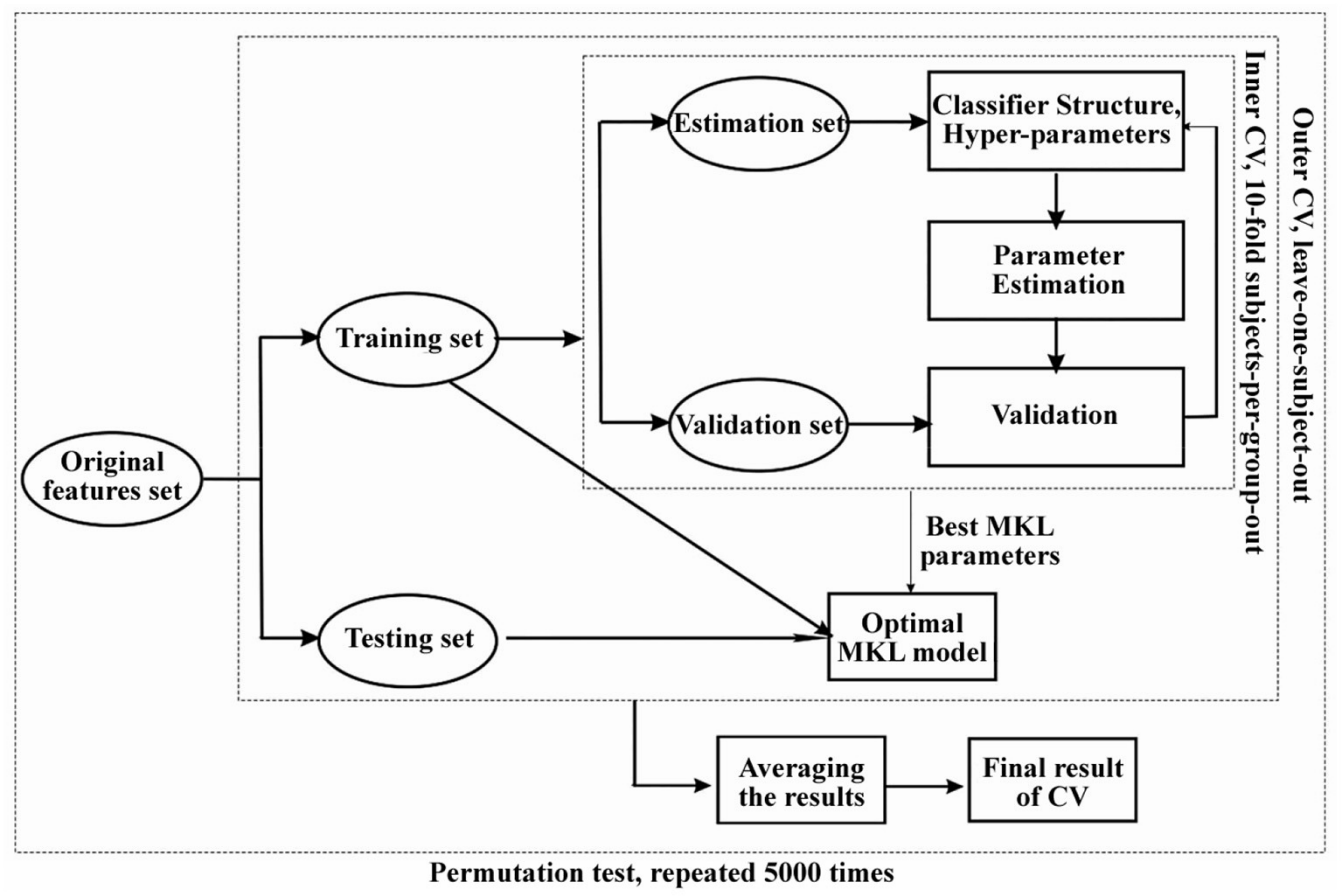
The flow chart of machine learning based multivariate pattern analysis (MVPA) and nested cross-validation pipeline used in our study. Permutation test was used to evaluate the significance of the classification performance for 5,000 times randomly.

Then, nested cross-validation (CV) with hyperparameter optimization was used to train the classification model and assess the generalization error (16). The outer loop was used to assess the model's performance, and the inner loop was used to optimize the model hyperparameters. For the inner loop, a 10-fold CV on subjects-per-group-out technique was used; for the outer loop, the leave-one-subject-out technique was used. As the simple MKL model employs a binary support vector machine (SVM) for classification, we used soft-margin hyperparameter optimization with the best configuration among C = 0.01, 0.1, 1 and 10. All C values were tested using a 10-fold CV (inner folds), and then the best C value was used for the outer loop (pipeline shown in Figure 2). As in our previous study (16), the statistics we used to evaluate classification performance are accuracy, sensitivity, specificity and the area under the curve (AUC) for the receiver operating characteristic (ROC) curve. Accuracy was defined as (TP+TN)/(TP+TN+FN+FP), where TP = true positive, TN =true negative, FP = false positive and FN = false negative. Sensitivity was defined as TP/(TP+FN), and specificity was defined as TN/(FP+TN).

After constructing the optimal classification model, we built weight maps representing the SVM weights per voxel and also maps summarizing the weights per ROI as defined by AAL atlas. As a simple kernel model was implemented by MKL-SVM, the weights per voxel will be averaged (in absolute value) within each region, and the regional weight maps were the spatial representation of the decision function that defined regional contributions to the classification process. Confidence intervals (*p*-values) generated by non-parametric permutation testing with 5000 randomizations were used to assure low variability in the outputs of classification models.

## Results

### Demographic and Clinical Characteristics

The demographic and clinical characteristics of each group are summarized in Table 1. One-way analysis of variance (ANCOVA) with LSD *post-hoc* tests was performed to assess differences in age, TIV, and MoCA, AVLT, SDMT, SCWT, and TMT scores, and a chi-square test was used to assess differences in sex, lacunes and vascular risk factors (smoking, hypertension, treated hypercholesterolemia and diabetes mellitus) among groups. The rank sum test of multiple independent samples (Kruskal-Wallis test) was used to compare CSVD scores and the severity of WMH among groups. The CSVD-c group had significantly lower MoCA, AVLT, and SDMT scores and significantly higher SCWT and TMT scores than the other groups. The CSVD-c group had a higher percentage of hypertension and treated hypercholesterolemia. No significant differences were found in age, sex, smoking and diabetes mellitus status, or TIV among the three groups.

**Table 1:** Demographic and clinical characteristics of CSVD patients and controls.

**Characteristics**	**CSVD-c**	**CSVD-n**	**HC**	***P*-value (ANOVA/χ^2^)**	***P*** **-value (** ***post-hoc*** **)**		
					**CSVD-c vs. HC**	**CSVD-c vs. CSVD-n**	**CSVD-n vs. HC**
Gender	16 M/10 F	21 M/22 F	17 M/22 F	0.359^χ^2	-	-	-
Age (y)	67.08 ± 6.19	66.68 ± 5.16	63.93 ± 8.87	0.105^a^	-	-	-
Education (y)	11.56 ± 2.81	11.33 ± 2.68	12.97 ± 3.53	0.041^a^			
smoking	10 (38.5%)	9 (20.9%)	10 (25.6%)	0.275^χ^2	-	-	-
Hypertension	24 (92.3%)	38 (88.3%)	12 (30.8%)	<0.001χ2	<0.001	0.600	<0.001
Treated Hypercholesterolaemia	15 (57.7%)	13 (30.2%)	5 (12.8%)	0.001^χ^2	0.024	<0.001	0.057
Diabetes Mellitus	9 (34.6%)	9 (20.9%)	4 (10.3%)	0.057^χ^2	-	-	-
Amended CSVD score	4 (2, 5.25)	2 (1, 2)	0 (0.0)	<0.001^b^	<0.001	<0.001	<0.001
WMH	2 (1, 3)	1 (1, 2)	0 (0.0)	<0.001^b^	<0.001	0.079	<0.001
Lacune	14 (53.8%)	3 (7.0%)	0 (0.0%)	<0.001^χ^2	<0.001	<0.001	0.093
MoCA	25.48 ± 2.67	27.58 ± 0.85	29.22 ± 3.27	<0.001^a^	<0.001	0.001	0.003
AVLT	54.80 ± 16.01	64.38 ± 9.02	67.83 ± 8.49	<0.001^a^	<0.001	<0.001	0.126
SDMT	23.96 ± 10.62	31.19 ± 7.55	41.95 ± 17.03	<0.001^a^	<0.001	0.016	<0.001
SCWT	186.67 ± 68.53	145.24 ± 26.82	134.77 ± 37.42	<0.001^a^	<0.001	<0.001	N.S.
TMT-A+B	332.92 ± 170.61	262.69 ± 74.01	208.58 ± 99.66	<0.001^a^	<0.001	0.012	0.032
TIV	1.61 ± 0.13	1.56 ± 0.14	1.62 ± 0.16	0.187^a^	-	-	-

χ*2: chi-square test*,

a *: one-way analysis of variance (ANOVA) test*,

b *: Kruskal-Walllis test. WMH, white matter hyperintensities. MoCA, Montreal Cognitive Assessment; AVLT, sum of Rey auditory verbal learning test (N1-7); SDMT, symbol digit modalities test; SCWT, sum of Stroop color-word test (stroop1-3); TMT, the trail-making test; TMT A+B, sum of TMT-A and TMT-B; TIV, total intracranial volume; CSVD-c, CSVD with CMBs group; CSVD-n, CSVD without CMBs group; HC, control group; N.S., not significant*.

### Univariate Analysis of GMV and WMV Differences

**Figure 3:**
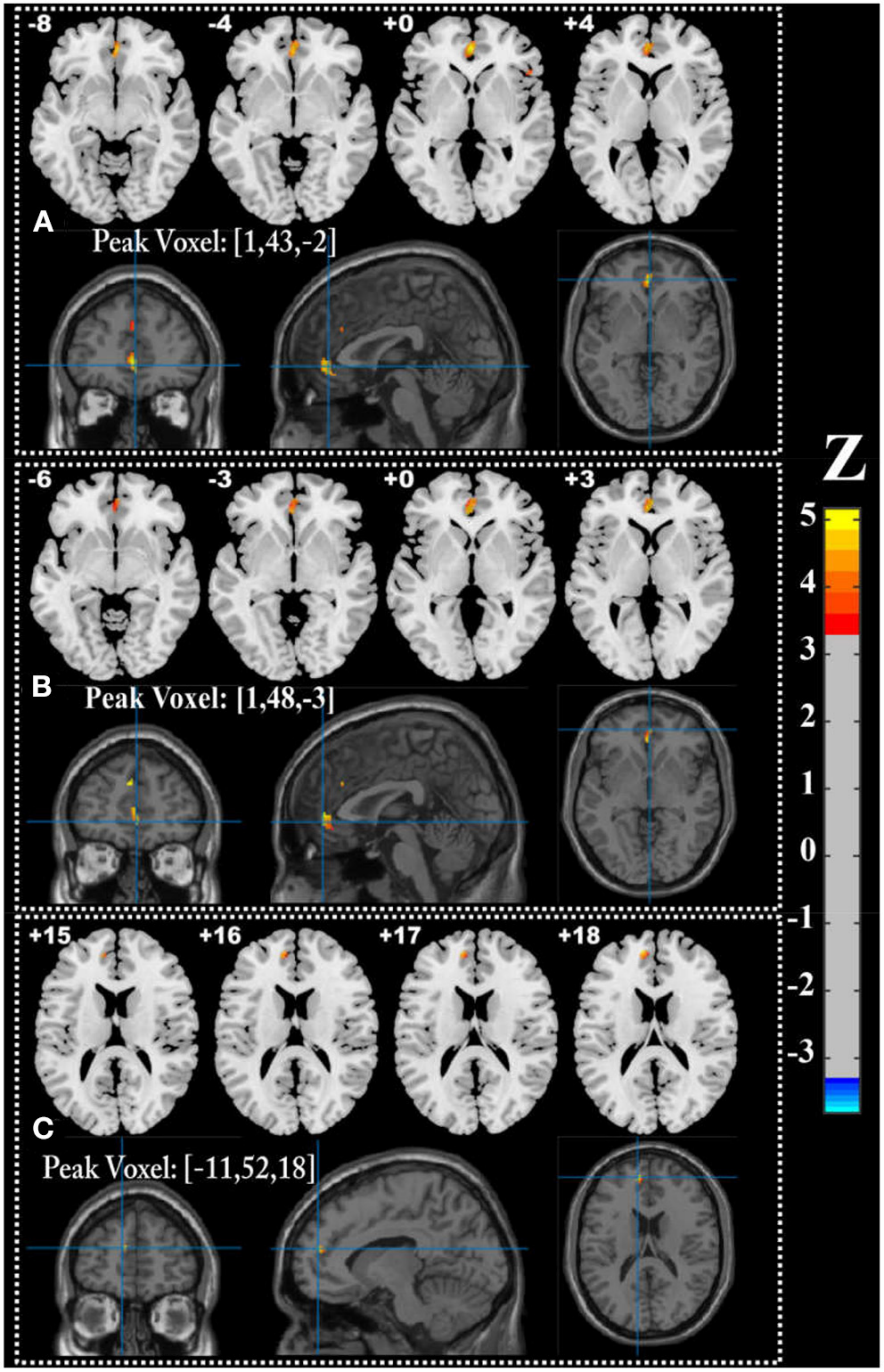
Brain regions showing significantly decreased GMV in **(A)** CSVD-c group and **(B)** CSVD-n group (ANOVA and LSD *post-hoc* test with GRF correction, voxel level *p* < 0.001, cluster level *p* < 0.05), and **(C)** decreased WMV in CSVD-n group compared with control group.

We performed univariate analyses to explore GMV and WMV alterations among groups. Compared with the control group, the CSVD-c group and CSVD-n group showed significantly (ANCOVA and LSD *post-hoc* test with GRF correction, voxel-level *p* < 0.001, cluster-level *p* < 0.05) decreased GMV in similar brain clusters, which mainly included the right superior frontal gyrus (medial orbital), left anterior cingulate gyrus, right inferior frontal gyrus (triangular part) and left superior frontal gyrus (medial). Meanwhile, the CSVD-n group also showed significantly decreased WMV in the cluster of the left medial superior frontal gyrus. The detailed results are shown in Table 2 and Figure 3. No significant differences in GMV or WMV were found between the CSVD-c group and the CSVD-n group.

**Table 2:** Significant altered GMV and WMV among three groups.

**Condition**	**Brain regions**	**Cluster size**	**z-score of peak voxel**	**MNI coordinates of peak voxel**		
				**x**	**y**	**z**
**GMV CSVD-c < control**	Right superior frontal gyrus, medial orbital	271	5.17	1	43	−2
	Right inferior frontal gyrus, triangular part	22	4.18	50	24	1
	Left anterior cingulate gyrus	16	4.16	1	33	29
	Left superior frontal gyrus, medial	38	3.80	−2	44	32
**GMV CSVD-n < control**	Right superior frontal gyrus, medial orbital	267	4.82	1	48	−3
	Left anterior cingulate gyrus	16	4.36	1	32	30
	Left superior frontal gyrus, medial	58	5.09	−2	44	32
**WMV CSVD-n < control**	Left superior frontal gyrus, medial	50	4.88	−11	52	18

*No significant differences were found between the two CSVD groups*.

*CSVD-c, CSVD with CMBs; CSVD-n, CSVD without CMBs*.

*Cluster size: the number of voxels in the (identified significant) cluster. ANOVA and LSD post-hoc test in a pair-wise manner within the areas identified by ANOVA were used to identify the GMV and WMV changes between groups with Gaussian random field (GRF) multiple comparison corrections (voxel level p <0.001, cluster level p <0.05)*.

### Multivariate Pattern Analysis and Classification Results

**Figure 4:**
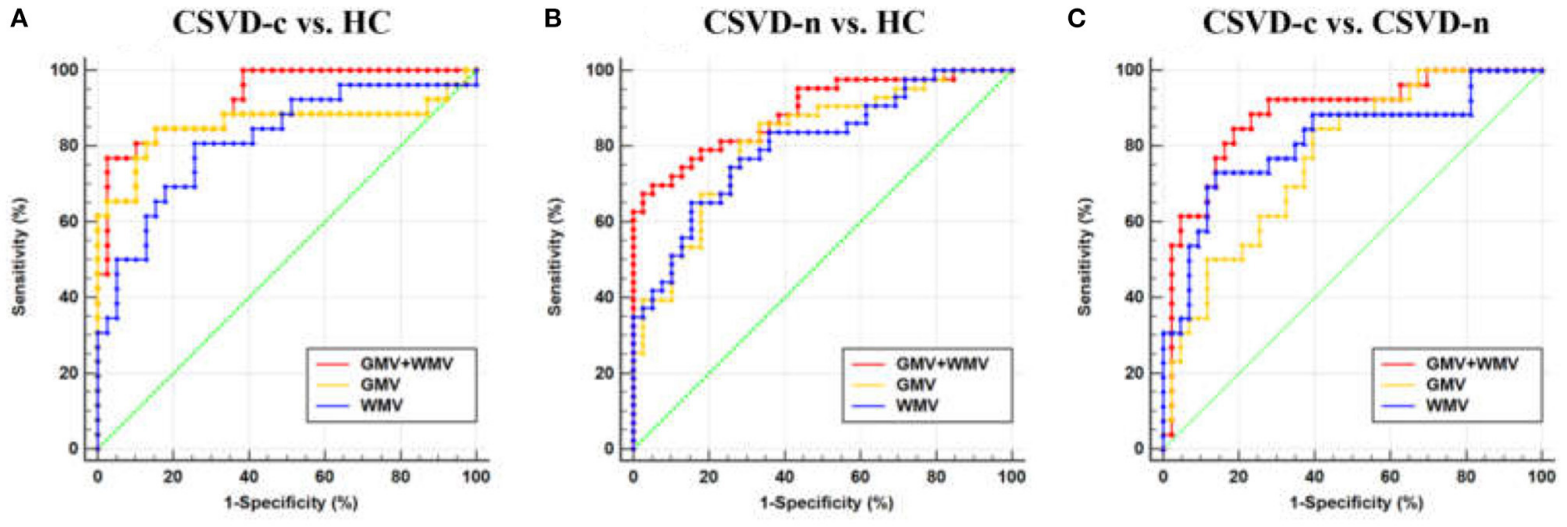
Receiver operating characteristic (ROC) curve for **(A)** CSVD-c vs. HC, **(B)** CSVD-n vs. HC, and **(C)** CSVD-c vs. CSVD-n classification problems. HC, healthy controls.

**Figure 5:**
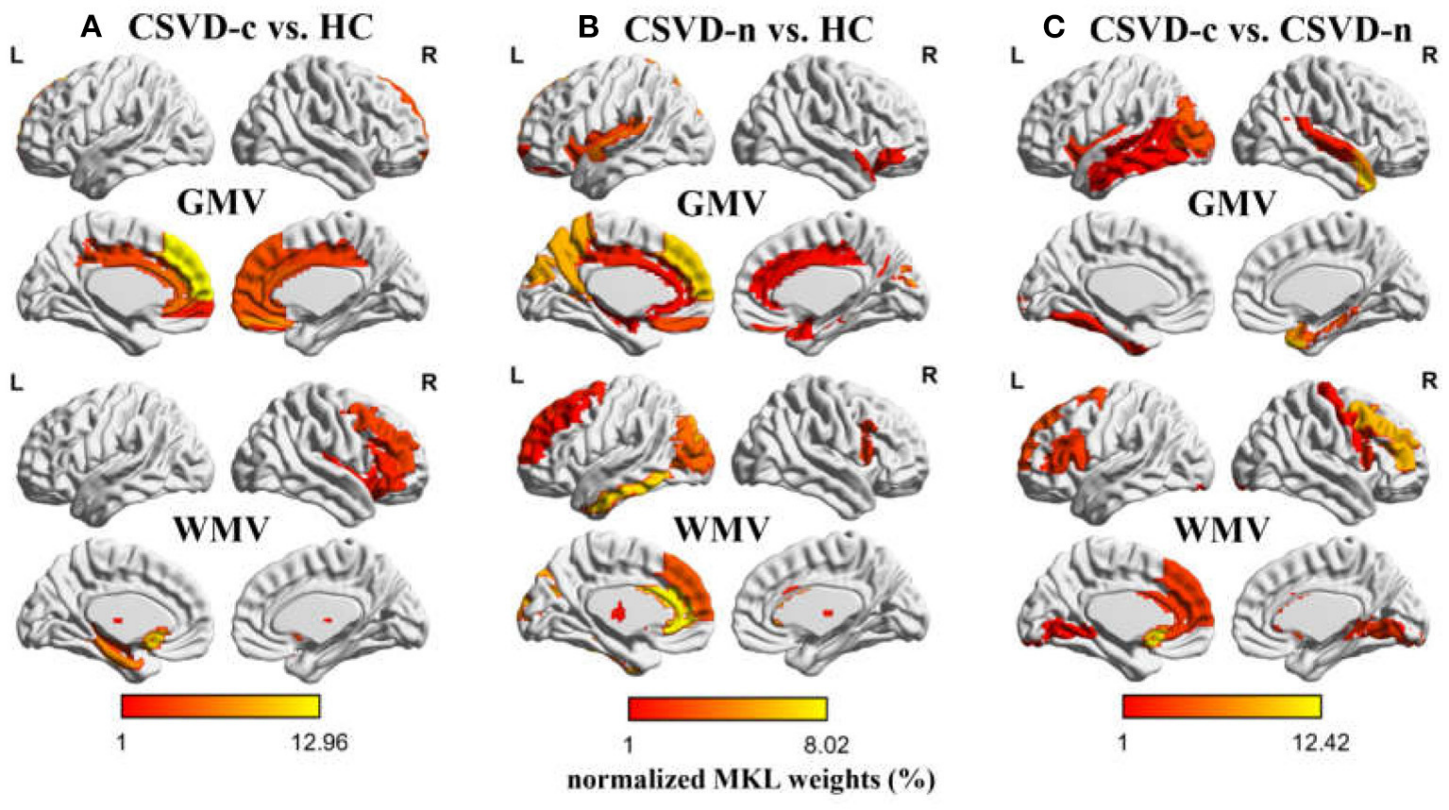
Weight (per region) maps modeled by multi-kernel learning (MKL) combining GMV and WMV features. As a simple kernel model was implemented by MKL-SVM, the weights per voxel will be averaged (in absolute value) within each ROI as defined by AAL atlas. The regional MKL weights representing regional contribution to **(A)** CSVD-c vs. HC, **(B)** CSVD-n vs. HC, and **(C)** CSVD-c vs. CSVD-n classification problems were rendered on the ICBM152 template. Weights with lower (1% or less) contribution are not shown. HC, healthy controls.

Regarding the three binary classification problems, the detailed statistics and ROC curves for evaluating classification performance are shown in Table 3 and Figure 4. As the results show, the MKL models that combined GMV and WMV features enhanced the classification accuracy for each binary classification problem. Based on the optimal MKL model combining GMV and WMV features, regional weight maps obtained by MKL (per region) were also calculated. The CSVD-c group vs. control group contrast suggested high regional weight in the bilateral medial superior frontal gyri (12.96 and 5.94%), bilateral anterior cingulate gyri (6.48 and 5.45%), right gyrus rectus (7.91%), and right superior frontal gyrus (medial orbital) (5.04%) for GMV and in the left olfactory cortex (12.09%) and left parahippocampal gyrus (7.91%) for WMV (Figure 5A). Meanwhile, the CSVD-n group vs. control group contrast suggested high regional weight in the left medial superior frontal gyrus (7.02%), cuneus (5.98%), and precuneus (5.96%) for GMV and in the left anterior cingulate gyrus (8.02%) and left inferior temporal gyrus (7.15%) for WMV (Figure 5B). In addition, the CSVD-c group vs. CSVD-n group contrast suggested high regional weight in the right temporal pole of the middle temporal gyrus (11.21%), right temporal pole of the superior temporal gyrus (7.39%), and right parahippocampal gyrus (5.62%) for GMV and in the left olfactory cortex (12.42%) and right middle frontal gyrus (9.50%) for WMV (Figure 5C). The ROIs with high weight detected from two CSVD groups vs. control group contrasts were relatively analogous to the ROIs with significant differences in the univariate analyses. Of note, we achieved good classification accuracy (81.16%) in the CSVD-c group vs. CSVD-n group, despite no significant differences between groups, and the ROIs with high weights in the MKL model provided important supplementary information for the univariate analyses.

**Table 3:** The statistics for evaluating classification performance.

**Modality**	**ACC**	**SEN**	**SPE**	**AUC**	**P**
**Condition:CSVD-c group vs. control group**					
**GMV**	84.62%	76.92%	89.74%	0.857	<0.05
**WMV**	76.92%	69.23%	82.05%	0.817	<0.05
**GMV+WMV**	86.15%	80.77%	89.74%	0.926	<0.05
**Condition:CSVD-n group vs. control group**					
**GMV**	74.39%	67.44%	82.05%	0.815	<0.05
**WMV**	74.39%	65.12%	84.62%	0.804	<0.05
**GMV+WMV**	81.71%	69.77%	94.87%	0.891	<0.05
**Condition:CSVD-c group vs. CSVD-n group**					
**GMV**	68.12%	57.69%	74.42%	0.770	<0.05
**WMV**	79.71%	73.08%	83.72%	0.815	<0.05
**GMV+WMV**	81.16%	69.23%	88.37%	0.881	<0.05

*ACC/SEN/SPE, accuracy/sensitivity/specificity; AUC, area under the ROC curve. P: p-values generated by non-parametric permutation testing with 5,000 randomizations*.

## Discussion

The current study applied both univariate analyses and the MVPA approach to explore brain morphological alterations in a relatively large cohort of CSVD patients with or without CMBs. Our study revealed that compared with the control group, the CSVD-c and CSVD-n groups showed significantly decreased GMV in similar brain clusters, which mainly included the right superior frontal gyrus (medial orbital), left anterior cingulate gyrus, right inferior frontal gyrus (triangular part) and left superior frontal gyrus (medial). According to many previous researchers, these regions with decreased GMV are mainly involved in the default mode network (DMN) (37). At the same time, the CSVD-n group showed significantly decreased WMV in the cluster of the left superior frontal gyrus (medial) compared with controls. No significant GMV or WMV differences were found between the CSVD-c group and the CSVD-n group. Additionally, CSVD patients could be differentiated from healthy controls using the MKL model based on GMV and WMV maps with high classification accuracy (86.15%; *p* < 0.05). Brain regions involving the DMN and subcortical regions were identified to have high differentiating power in these MKL modles. Meanwhile, we achieved good classification accuracy (81.16%) in the CSVD-c group vs. CSVD-n group contrast using the MKL model.

Using univariate analyses, our results showed that the CSVD-c group and CSVD-n group had significantly decreased GMV in similar brain clusters compared with the healthy control group, mainly involving the DMN. The core mechanism underlying CSVD-related brain injury is diffuse cerebrovascular endothelial failure. Endothelial damage leads to increased permeability with leakage of material into the vessel wall and perivascular tissue, leading to brain inflammation (38). Brain inflammation can lead to neuronal dysfunction and cell death (39, 40). In this study, we obtained similar results. We found that the TMT scores were significantly higher and the MoCA, AVLT and SDMT scores were significantly lower in the CSVD patients than in the healthy controls, suggesting that these related cognitive functions were significantly disrupted in CSVD patients. We thought this might be because the decreased GMV regions associated with CSVD mainly involved the DMN. The DMN is a set of functionally connected regions that plays crucial roles in internal cognitive processing. DMN connectivity strength has been positively correlated with individual cognitive performances (e.g., working memory, autobiographical memory, attention, and language) (41), and damage to the default network can cause cognitive impairment.

Compared to traditional univariate analysis, the MVPA approach can be used to make diagnostic decisions. Our results showed that the combination of GMV and WMV features improved the accuracy for each binary classification problem compared to using GMV or WMV features alone. For the classification of the CSVD and control groups, the high-weighted GMV feature areas included the bilateral superior frontal gyrus (medial part or medial orbital part) and bilateral anterior cingulate gyrus. More volume loss regions were identified compared to the univariate analyses. Meanwhile, many high-weighted WMV feature regions could also be found, such as the left anterior cingulate gyrus, left para-hippocampal gyrus and left inferior temporal gyrus.

The anterior cingulate gyrus is one of the important structures responsible for the executive function of the brain, and it mainly monitors ongoing directional behaviors and coordinates cognitive processes (42). The medial part of the superior frontal gyrus is commonly deactivated during the cognitive-related processing and has been ascribed to be a component of the default mode network and a study also found that the superior frontal gyrus is anatomically connected with the cingulate cortex (mostly the anterior cingulate gyrus and the mid-cingulate cortex) through resting-state functional connectivity analysis (43). These results suggest the injury of superior frontal gyrus and anterior cingulate gyrus related to decline of cognitive control. Our results also indicated that the impaired anterior cingulate gyrus and superior frontal gyrus were related with the decline of cognitive function. A study revealed damaged GMV and functional connectivity (FC) in the cerebellum in CSVD patients with WMH and reduced connectivity of cerebellar lobule VI to the left anterior cingulate gyrus owing to WMH (44). These results, similar to ours, showed WM lesions in CSVD. Furthermore, WMH can disrupt white matter tracts or U-fibers that mediate cortical–cortical or cortical–subcortical connections (45), which also provides evidence for our results.

The results of previous studies were inconsistent in the relationship between CMBs and WMV morphological changes, one showed reduced WMV (14), the other showed increased WMV (15). But using the MVPA approach could overcome this shortcoming came from the embarrassment of different conclusions, our analysis just concern existential WM changes, no matter WMV reduced or increased. Our results showed that CSVD should be considered a whole-brain disease and that GMV and WMV were both significantly altered, which was also hinted at by previous studies indicating that local white matter lesions may influence the gray matter in remote areas (46). Therefore, we combined between-group statistical comparison and MVPA-based individual classification to provide complementary information for revealing the potential pathogenesis of CSVD and assisting clinical diagnosis.

Notably, no significant GMV or WMV difference was found in univariate analysis between the CSVD-c and CSVD-n groups. This might reveal that the presence or absence of cerebral CMBs in patients with CSVD has little effect on changes in GMV, which is consistent with the findings of previous research (15). However, the MVPA analysis could mine multivoxel spatial pattern information and achieved a relatively good classification accuracy between the two CSVD groups (81.16%; *p* < 0.05). The classification of the CSVD-c and CSVD-n groups suggested that high-weighted regions were in the right temporal pole of the middle temporal gyrus, temporal pole of the superior temporal gyrus, right para-hippocampal gyrus for GMV and in the left olfactory cortex and right middle frontal gyrus for WMV. This result might have reflected more potential WM and GM changes in the CSVD-c group than in the CSVD-n group. CSVD-c patients have more severe inflammation in the brain because CMBs themselves could lead to a sustained local inflammatory response, characterized by initial activation and persistent increase in microglia and macrophages (47). Many investigations have reported that CMBs are associated with cognitive dysfunction in the elderly (48–50), and epidemiological studies have shown that CMBs adversely affect the cognitive function of patients with CSVD and are independent risk factors for cognitive decline (51). In our study, the CSVD-c patients had more serious cognitive problems than the CSVD-n patients. Hence, we speculated that more severe brain inflammation problems in CSVD-c patients might be the reason for them having poor cognitive scores. The middle temporal gyrus has been shown to be recruited during the processing of words and during the observation of actions (52). The human superior temporal gyrus is critical for extracting meaningful linguistic features from speech input (53). The hippocampus and surrounding medial temporal lobe structures play a key role in learning and memory formation (54). The hippocampus and parahippocampus were associated with olfactory ability (55). These previous studies showed the involved regions in CSVD-c patients correlated with cognitive function. Therefore, our results hinted the injury of right temporal lobe, right para-hippocampal gyrus and the left olfactory might be the reason why CSVD-c group had more serious congnitive decline.

In this study, we conducted VBM-DARTEL preprocessing using VBM8 within SPM8 toolbox. A recent study (56) has shown that VBM analysis through CAT12 within SPM12 toolbox is more robust to detect the small brain morphological changes than VBM8. Therefore, we also conducted VBM-DARTEL preprocessing using CAT12 as the supplementary experiment. The univariate results showed the number of significant voxels revealed by CAT12 method is more than VBM8 method, while the structural localization of peak voxels is similar between these two methods, and detailed results were shown in Supplementary Materials. In our future research, we will combine VBM and MVPA analysis based on CAT12. More longitudinal studies with a large sample size are needed in the future to understand the relationship between CMBs and the cerebral morphology changes. Besides, more specific features for neurodegeneration such as age, gender and cardiovascular risk factors should be included in future research to explore the neurodegeneration pathology of CSVD.

## Conclusion

In conclusion, we found significantly lower decreased GMV and WMV in the frontal and anterior cingulate gyrus regions in CSVD patients. In addition, our work has shown that an appropriate a proper combination of MVPA and MKL methods can substantially improve the classification accuracy of CSVD patients. Our findings may clarify the potential pathogenesis of CSVD and provide further support in favor of machine-learning approaches in improving the clinical diagnosis of CSVD.

## Data Availability Statement

The original contributions presented in the study are included in the article/Supplementary Material, further inquiries can be directed to the corresponding author.

## Ethics Statement

The studies involving human participants were reviewed and approved by the Institutional Review Board of Shandong Provincial Hospital Affiliated to Shandong First Medical University. The patients/participants provided their written informed consent to participate in this study.

## Author Contributions

JL and HW wrote the main manuscript text. HW prepared Figures 1–5. SW, YC, and NZ prepared the clinical data and imaging data. LG revised the main manuscript text. All authors reviewed the manuscript.

## Funding

This work was supported by grants from the National Natural Science Foundation of China (81800840), the National Natural Science Foundation of China (32100902), the Fundamental Research Funds for the Central Universities (SWU118065), the Technology Development Plan of Jinan (201301049, 201602206, and 201907052), Medical and Health Science and Technology Development Project of Shandong Province (2016WS0529 and 2019WS544), Funding for Study Abroad Program by Shandong Province (201803059), and Shandong Provincial Natural Science Foundation (ZR2020MH288).

## Conflict of Interest

The authors declare that the research was conducted in the absence of any commercial or financial relationships that could be construed as a potential conflict of interest.

## Publisher's Note

All claims expressed in this article are solely those of the authors and do not necessarily represent those of their affiliated organizations, or those of the publisher, the editors and the reviewers. Any product that may be evaluated in this article, or claim that may be made by its manufacturer, is not guaranteed or endorsed by the publisher.
