# Supplementary material for: Altered Brain Morphometry in Cerebral Small Vessel Disease With Cerebral Microbleeds: An Investigation Combining Univariate and Multivariate Pattern Analyses
Source: Front Neurol. 2022 Feb 23;13:819055. doi: 10.3389/fneur.2022.819055 (PMC8904567; doi:10.3389/fneur.2022.819055)
Supplement: Supplementary file 1 [file Table_1.DOCX]

**Materials and Methods**

**VBM-DARTEL processing using CAT 12**

In primary analysis, VBM-DARTEL processing was conducted using VBM8 toolbox within SPM8 software. As the supplementary experiment, we also conducted VBM-DARTEL processing using CAT12 toolbox (http://www.neuro.uni-jena.de/cat12-html/cat_versions.html) within SPM12 software. Briefly, the same processing pipeline including C-PC alignment, segmenting aligned images into GM, WM, and CSF components, DARTEL normalization, modulation, smoothing with Gaussian kernel of 8-mm FWHM, were implemented via the VBM8 and CAT12 toolboxes, respectively. Of note, the main improvements of VBM processing in SPM12 were normalization and segmentation methods compared to the older program SPM8, but the DARTEL process of normalizing to an averaged group template is not updated in SPM12.

Next, the total GM volume, WM volume, and CSF volume were obtained, separately, on the basis of segmented images. The total intracranial volume (TIV) was calculated as the sum of the GM, WM, and CSF volumes for each toolbox, separately. To compare GMV and WMV and identify abnormalities among the three groups, the same design (ANCOVA with LSD post-hoc tests, controlling age, sex and TIV as covariates) is used for univariate analysis based on VBM8 and CAT12 respectively.

**ROI defined based on AAL atlas in Multivariate pattern analysis**

We used the automated anatomical labeling (AAL) template to divide the entire brain into 90 cortical and subcortical regions (45 for each hemi-sphere), and the detailed information for 90 ROIs was shown in Table S1.

Table S1. The 90 cortical and subcortical ROIs (45 for each hemi-sphere) defined in our study.

| Regions | Abbr. | Regions | Abbr. |
| --- | --- | --- | --- |
| Precentral gyrus | PreCG | Lingual gyrus | LING |
| Superior frontal gyrus, dorsolateral | SFGdor | Superior occipital gyrus | SOG |
| Superior frontal gyrus, orbital part | ORBsup | Middle occipital gyrus | MOG |
| Middle frontal gyrus | MFG | Inferior occipital gyrus | IOG |
| Middle frontal gyrus orbital part | ORBmid | Fusiform gyrus | FFG |
| Inferior frontal gyrus, opercular part | IFGoperc | Postcentral gyrus | PoCG |
| Inferior frontal gyrus, triangular part | IFGtriang | Superior parietal gyrus | SPG |
| Inferior frontal gyrus, orbital part | ORBinf | Inferior parietal, but supramarginal and angular gyri | IPL |
| Rolandic operculum | ROL | Supramarginal gyrus | SMG |
| Supplementary motor area | SMA | Angular gyrus | ANG |
| Olfactory cortex | OLF | Precuneus | PCUN |
| Superior frontal gyrus, medial | SFGmed | Paracentral lobule | PCL |
| Superior frontal gyrus, medial orbital | ORBsupmed | Caudate nucleus | CAU |
| Gyrus rectus | REC | Lenticular nucleus, putamen | PUT |
| Insula | INS | Lenticular nucleus, pallidum | PAL |
| Anterior cingulate and paracingulate gyri | ACG | Thalamus | THA |
| Median cingulate and paracingulate gyri | DCG | Heschl gyrus | HES |
| Posterior cingulate gyrus | PCG | Superior temporal gyrus | STG |
| Hippocampus | HIP | Temporal pole: superior temporal gyrus | TPOsup |
| Parahippocampal gyrus | PHG | Middle temporal gyrus | MTG |
| Amygdala | AMYG | Temporal pole: middle temporal gyrus | TPOmid |
| Calcarine fissure and surrounding cortex | CAL | Inferior temporal gyrus | ITG |
| Cuneus | CUN |  |  |

**Results**

**Univariate analysis of GMV and WMV differences based on CAT12**

In the supplementary experiment, compared with the control group, the CSVD-c group and CSVD-n group showed significantly (ANCOVA and LSD post hoc test with GRF correction, voxel-level p<0.001, cluster-level p<0.05) decreased GMV in similar brain clusters, which mainly included the right superior frontal gyrus (medial orbital), left anterior cingulate gyrus, right frontal gyrus WM and right amygdala. Meanwhile, the CSVD-n group also showed significantly decreased WMV in the cluster of the left medial superior frontal gyrus, left thalamus and parahippocampal gyrus. The detailed results are shown in Table S2 and Figure S1. No significant differences in GMV or WMV were found between the CSVD-c group and the CSVD-n group.

**Table S2.** Results of univariate analysis based on CAT12. ANOVA and LSD post-hoc tests were used to identify the GMV and WMV changes among groups with Gaussian random field (GRF) multiple comparison corrections (voxel level p<0.001, cluster level p<0.05).

| Condition | Brain  regions | Cluster  size | z-score of  peak voxel | MNI coordinates of peak voxel | | |
| --- | --- | --- | --- | --- | --- | --- |
|  |  |  |  | x | y | z |
| GMV  CSVD-c<control | Left anterior cingulate gyrus/Right superior frontal gyrus, medial orbital | 664 | 5.17 | -1 | 44 | 2 |
|  | Right frontal gyrus WM/Right Amygdala | 149 | 4.19 | 23 | 8 | -14 |
| GMV  CSVD-n<control | Right superior frontal gyrus, medial orbital/Left anterior cingulate gyrus | 580 | 5.30 | 0 | 44 | 4 |
|  | Right frontal gyrus WM/Right Amygdala/ Right parahippocampal gyrus | 222 | 4.82 | 21 | 4 | -18 |
| WMV  CSVD-n<control | Left superior frontal gyrus, medial | 38 | 4.45 | -11 | 52 | 18 |
|  | Left thalamus | 70 | 3.95 | -15 | -16 | 3 |
|  | Left parahippocampal gyrus | 22 | 3.73 | -15 | -29 | -11 |

Abbreviations: CSVD-c, CSVD with CMBs; CSVD-n, CSVD without CMBs.


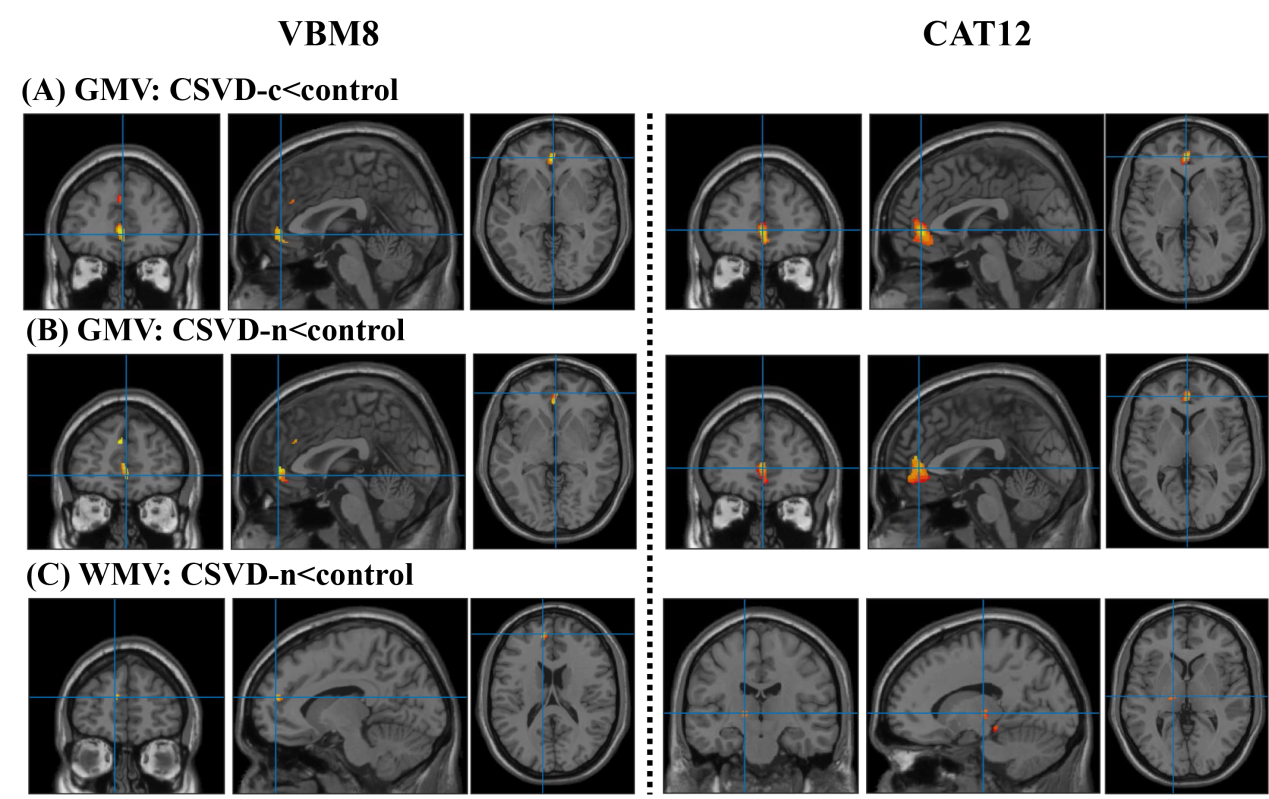


**Figure S1.** The significant alterations of regional GMV and WMV revealed by the voxel-based morphometry (VBM) analyses using VBM8 versus CAT12.
